# Inhibition of the endosymbiont “*Candidatus* Midichloria mitochondrii” during 16S rRNA gene profiling reveals potential pathogens in *Ixodes* ticks from Australia

**DOI:** 10.1186/s13071-015-0958-3

**Published:** 2015-06-25

**Authors:** Alexander W. Gofton, Charlotte L. Oskam, Nathan Lo, Tiziana Beninati, Heng Wei, Victoria McCarl, Dáithí C. Murray, Andrea Paparini, Telleasha L. Greay, Andrew J. Holmes, Michael Bunce, Una Ryan, Peter Irwin

**Affiliations:** Vector and Water-Borne Pathogen Research Laboratory, School of Veterinary and Life Sciences, Murdoch University, Perth, Western Australia Australia; School of Biological Sciences, The University of Sydney, Sydney, New South Wales Australia; Faculty of Veterinary Science, The University of Sydney, Sydney, New South Wales Australia; Trace and Environmental DNA Laboratory, Department of Environment and Agriculture, Curtin University, Perth, Western Australia Australia; School of Molecular Biosciences and Charles Perkins Centre, The University of Sydney, Sydney, New South Wales Australia

**Keywords:** Tick, Vector-borne disease, Zoonoses, Metagenomics, 16S community profiling, *Ixodes holocyclus*, *Ixodes ricinus*, *Candidatus* Midichloria, *Borrelia*, *Candidatus* Neoehrlichia

## Abstract

**Background:**

The Australian paralysis tick (*Ixodes holocyclus*) is of significant medical and veterinary importance as a cause of dermatological and neurological disease, yet there is currently limited information about the bacterial communities harboured by these ticks and the risk of infectious disease transmission to humans and domestic animals. Ongoing controversy about the presence of *Borrelia burgdorferi* sensu lato (the aetiological agent of Lyme disease) in Australia increases the need to accurately identify and characterise bacteria harboured by *I. holocyclus* ticks.

**Methods:**

Universal PCR primers were used to amplify the V1-2 hyper-variable region of bacterial 16S rRNA genes present in DNA samples from *I. holocyclus* and *I. ricinus* ticks, collected in Australia and Germany respectively. The 16S amplicons were purified, sequenced on the Ion Torrent platform, and analysed in USEARCH, QIIME, and BLAST to assign genus and species-level taxonomy. Initial analysis of *I. holocyclus* and *I. ricinus* identified that > 95 % of the 16S sequences recovered belonged to the tick intracellular endosymbiont “*Candidatus* Midichloria mitochondrii” (CMM). A CMM-specific blocking primer was designed that decreased CMM sequences by approximately 96 % in both tick species and significantly increased the total detectable bacterial diversity, allowing identification of medically important bacterial pathogens that were previously masked by CMM.

**Results:**

*Borrelia burgdorferi* sensu lato was identified in German *I. ricinus*, but not in Australian *I. holocyclus* ticks. However, bacteria of medical significance were detected in *I. holocyclus* ticks, including a *Borrelia* relapsing fever group sp., *Bartonella henselae,* novel “*Candidatus* Neoehrlichia” spp., *Clostridium histolyticum*, *Rickettsia* spp., and *Leptospira inadai*.

**Conclusions:**

Abundant bacterial endosymbionts, such as CMM, limit the effectiveness of next-generation 16S bacterial community profiling in arthropods by masking less abundant bacteria, including pathogens. Specific blocking primers that inhibit endosymbiont 16S amplification during PCR are an effective way of reducing this limitation. Here, this strategy provided the first evidence of a relapsing fever *Borrelia* sp. and of novel “*Candidatus* Neoehrlichia” spp. in Australia. Our results raise new questions about tick-borne pathogens in *I. holocyclus* ticks.

**Electronic supplementary material:**

The online version of this article (doi:10.1186/s13071-015-0958-3) contains supplementary material, which is available to authorized users.

## Background

Ticks are the second most important vector of pathogens to humans after mosquitoes and the chief cause of vector-borne diseases in domestic animals and wildlife [1–3]. Ticks also vector the greatest diversity of pathogenic microorganisms of any haematophagous arthropod, including members of the bacterial genera *Anaplasma* [4]*, Bartonella* [5]*, Borrelia* [6], *Ehrlichia* [7], *Francisella* [8]*, Rickettsia* [9], and “*Candidatus* Neoehrlichia” [10]. Furthermore, bacterial co-infections in ticks are common and provide diagnostic and therapeutic challenges for medical and veterinary practitioners [11–13]. In Europe, North America, and Australia the incidence of tick-borne diseases is rising due to a combination of factors including perturbation in climate, increasing populations and movement of humans and domestic animals, and increased human encroachment into tick habitats [14].

In Australia there is a long-standing controversy concerning the presence of Lyme disease and its aetiological agents, *Borrelia burgdorferi* sensu lato. First reported in the 1980s [15, 16], intensive efforts to determine the aetiological agent of Australian “Lyme-like” illness found no evidence for *B. burgdorferi* sensu lato in ticks or wildlife [17, 18], yet numerous victims of tick bites continue to present with Lyme-like symptoms in Australia [19]. Thus there is a pressing need to apply contemporary next-generation sequencing (NGS) techniques to better understand bacterial pathogens harboured in Australian ticks.

In Australia *I. holocyclus* is the most important tick species from both a medical and veterinary perspective [20, 21]. Its enzootic range is limited to a narrow strip along Australia’s eastern seaboard that extends several thousand kilometres from Cape York to eastern Victoria, and includes most of Australia’s most densely populated regions [22]. *Ixodes holocyclus* is commonly found on domestic animals in which it causes life-threatening paralysis. *Ixodes holocyclus* is also the most common tick found on people in its range and impacts human health by causing weakness, paralysis, allergic reactions, and is a vector for the spotted fever pathogens *Rickettsia australis* and *R. honei* [23].

Together with known vector-borne pathogens, ticks also harbour closely related endosymbiotic bacteria such as *Coxiella* spp*.* [24–26], *Francisella* spp. [27–29], *Wolbachia* spp. [30, 31], *Rickettsia* spp. [32–35], and the recently discovered “*Candidatus* Midichloria mitochondrii” (CMM) [36–39]. These bacterial endosymbionts often dominate the microbial population within their arthropod hosts and can affect the transmission dynamics of pathogenic species [40–42].

CMM is an intracellular endosymbiont that was first discovered in the European sheep tick *Ixodes ricinus* [36] but has since been detected in other ticks including *I. holocyclus* [37, 43–47], as well as tabanid flies [48], bed bugs [49], and mites [50]. In ticks, CMM resides in high numbers in female reproductive tissues and is transmitted to all offspring where it infects 100 % of larvae, nymphs, and females [36, 51]. Male *I. holocyclus* ticks also appear to inherit and harbour CMM, however, *I. ricinus* males fail to establish stable CMM populations [36, 38, 51]. In addition to this, CMM is found in *I. ricinus* salivary glands from where it is introduced during feeding to vertebrate hosts, including humans [52, 53]. However, the consequences of CMM infection in vertebrate hosts, if any, are unknown [52].

Next-generation sequencing and bioinformatics advances have greatly increased our ability to accurately identify trace amounts of DNA in highly heterogeneous samples, making them excellent tools for molecular epidemiological studies of pathogens that may be present in low abundance. In particular, the application of 16S rRNA gene (hereafter referred to as 16S) community profiling has been particularly successful for characterising bacterial assemblages from a wide variety of sources, including ticks [30, 54–62]. With this methodology, a short region (100-500 bp) of the 16S gene is amplified using PCR primers that bind to orthologous regions either end of a hyper-variable region of the gene. Because the primers bind to orthologous regions of the 16S gene numerous bacterial taxa within a heterogeneous sample can be targeted simultaneously, and the hyper-variable region proximal to the primers permits taxonomic discrimination of those taxa [63, 64].

A limitation of 16S community profiling in ticks is that a high proportion of sequences generated during PCR will belong to bacterial endosymbionts [40]. These overabundant endosymbiont 16S sequences can mask the presence of less abundant bacterial 16S sequences including pathogens, resulting in biased results and a decreased detected bacterial diversity. This limitation can be overcome to some extent by deeper sequencing to increase detection of low abundant sequences. However, this approach fails to address the source of the problem and is costly, making it difficult to study a large number of samples. In addition, various factors such as biases in PCR amplification efficiency and inter-specific variation of the 16S copy number are known to skew the measured proportion of NGS reads, and limits the use of sequence abundance to infer actual bacterial abundance in the original sample [65, 66].

As part of an ongoing study into tick-borne diseases in Australia, we developed a primer that inhibits amplification of CMM 16S sequences, enabling us to identify other less abundant bacteria in *I. holocyclus* and *I. ricinus*. This approach has provided insights into the bacterial microbiome of *I. holocyclus* and is readily applicable to other arthropod vectors of plant and animal diseases where overabundant species prove problematic to the identification of important taxa.

## Methods

### Sample collection

A total of 196 individual specimens of *I. holocyclus*, were collected from mammalian (*n =* 85) and avian (*n =* 2) hosts, and from the environment (*n =* 109) in various locations in New South Wales, Australia, between 2004 and 2014 (Table 1). All host-seeking *I. holocyclus* ticks were collected by flagging, using standard techniques [67], and either preserved frozen, stored in 70 % ethanol, or used immediately. In addition, 20 nymph and ten female *I. ricinus* ticks were collected by flagging in suburban parks in the cities of Freising and Leipzig, Germany, in 2013, and were immediately placed in 70 % ethanol and shipped to Murdoch University. All ticks were identified morphologically using standard keys [68, 69].

**Table 1:** *Ixodes holocyclus* and *I. ricinus* ticks collected from different hosts and the environment

Tick Instar or Sex	Number of ticks	Hosts or Questing (number of ticks)
*Ixodes holocyclus*	196	
Nymph	15	Questing (15)
Male	41	Questing (41)
Female	140	*Bos taurus* (4), *Canis familiaris* (26), *Corvus coronoides* (1), *Cracticus tibicen* (1), Echidna (Family: *Tachyglossidae*) (1), *Felis catus* (5), *Homo sapiens* (26), *Macropus spp.* (9), *Trichosurus vulpecula* (14), Questing (53).
*Ixodes ricinus*	30	
Nymph	20	Questing (20)
Female	10	Questing (10)

### Ethics statement

This research complies with the *Australian Code for the Responsible Conduct of Research, 2007* and the *Australian Code for the Care and Use of Animals for Scientific Purposes, 2013*. Removal of ticks from animal hosts was approved by the Murdoch University Animal Ethics Committee; collection from domestic animals (*n* = 35) and wildlife species (*n* = 26) was opportunistic, from individuals that were presented to veterinarians, or were dead as a result of unrelated accident or injury. Ticks (*n* = 26) were removed from humans by the person themselves or by medical professionals during outpatient treatment.

### DNA extraction

Total genomic DNA from individual ticks was extracted using the Qiagen DNeasy Blood and Tissue Kit (Qiagen, Germany) following the manufacturer’s recommendations (Qiagen Supplementary Protocol: Purification of total DNA from insects). Before extraction, individual ticks were surface sterilised in 10 % sodium hypochlorite, washed in 70 % ethanol and DNA-free PBS, frozen in liquid nitrogen for 1 min, and homogenised with 5 mm steel beads in a Tissue Lyser LT (Qiagen, Germany) for 1 min at 40 Hz. DNA-free equipment and tubes were used for each step and equipment was decontaminated between samples with DNAaway (Life Technologies, USA). Extraction reagent blanks were performed in parallel with all DNA extractions in order to determine background bacterial populations (one extraction reagent blank for every 23 samples). To prevent potential cross-contamination by known *I. ricinus* pathogens, DNA extractions from these ticks were performed in a separate laboratory to *I. holocyclus* DNA extractions.

### Blocking primer design

**Fig. 1:**
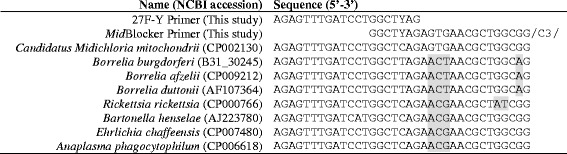
Alignment of partial 16S rDNA sequences and the 27F-Y and *Mid*Blocker primers. Alignment includes partial 16S sequences of seven tick-borne bacterial pathogens and “*Candidatus* M. mitochondrii” with the 27F-Y and *Mid*Blocker primers showing mismatches that allow specific blocking of “*Candidatus* M. mitochondrii”

In pilot 16S community profiling experiments, over 95 % of the sequences generated from each sample, from both *I. holocyclus* and *I. ricinus* ticks, belonged to CMM regardless of the sequencing depth, PCR primers, or sequencing platform used (data not shown). To inhibit amplification of these overabundant sequences during PCR, we developed a CMM-specific blocking primer (*Mid*Blocker) [70] to be used in conjunction with the 16S universal primers 27F-Y (Fig. 1) and 338R (5’-TGCTGCCTCCCGTAGGAGT-3’) that amplify the V1-V2 16S region [71]. The *Mid*Blocker primer was designed from an alignment of 107 partial 16S sequences including known tick-borne pathogens and endosymbionts, ubiquitous environmental bacteria, and CMM (Fig. 1)*.* The 5’ end of the *Mid*Blocker primer has a 7 bp overlap with the 3’ end of the 27F-Y primer, extends 15 bp downstream of the 27F-Y primer-binding site, and terminates polymerase elongation due to a C3 spacer at the 3’ end of the primer (Fig. 1). *In silico* analysis (not shown) suggests that the *Mid*Blocker primer is specific to CMM and will not modulate the binding of the 27F-Y primer to other closely related Rickettsiaceae and Anaplasmataceae.

### Validation of the *Mid*Blocker primer

Total DNA from host-seeking female *I. holocyclus* (*n* = 10) and *I. ricinus* (*n* = 10) were amplified by qPCR using the 27F-Y and 338R primers with and without 10 μM of *Mid*Blocker primer. Different concentrations (2-14 μM) of the *Mid*Blocker primer were trialled in pilot experiments on a subset of samples (data not shown). PCR conditions, fusion-primer architecture, semiconductor sequencing, and sequence analysis were the same as described below. Nonparametric Mann-Whitney U-tests were performed in Quantitative Insights Into Microbial Ecology (QIIME) [72] to determine the significance of differences in bacterial diversity between samples amplified with and without *Mid*Blocker; significance was set at *p* < 0.05 (Mann-Whitney U-Test).

### 16S community profiling qPCR

The primers 27F-Y and 338R amplified the 16S V1-2 hyper-variable regions (250-320 bp) [73] in *I. holocyclus* and *I. ricinus* DNA samples. 27F-Y and 388R primers also incorporated a six to eight base pair multiplex identifier (MID) sequence together with Ion Torrent sequencing adapters A and P1 (Life Technologies, USA). Each sample was amplified with primers containing a unique combination of forward and reverse MID sequences to allow multiplex sequencing and discrimination of sequences to samples in downstream analysis. All community profiling qPCRs were carried out in duplicate in 25 μl reactions containing 1 × PCR buffer (5 prime, Germany), 2 mM MgCl_2_ (5 Prime, Germany), 0.25 mM dNTPs (Fisher Biotech, Australia), 0.01 mg BSA (Fisher Biotech, Australia), 0.4 μM of each 27F-Y and 338R primer, 10 μM of *Mid*Blocker, 0.12 × SYBR Green (Life Technologies, USA), 1 U of Perfect Taq Polymerase (5 Prime, Germany), 1 × ROX dye (Life Technologies, USA), and 2 μl of DNA (1-100 ng/μl). No-template control reactions and extraction reagent blank controls were included in every qPCR run and were incorporated in the sequencing libraries. All PCR amplifications were performed on a Step-One real-time qPCR machine (Applied Biosystems, USA) with the following thermal conditions: initial denaturation at 95 °C for 5 min followed by 35 cycles of denaturation at 95 °C (30s), annealing at 62 °C (30s), and extension at 72 °C (45 s). Thermocycling was followed by a melt curve and final extension at 72 °C for 10 min.

### Library preparation and NGS

16S amplicons from all samples and controls were pooled into one of four sequencing libraries in equimolar amounts. Amplicon libraries were then purified twice using 1.2 volumes of Agencourt Ampure XP beads (Agilent Technologies, USA) and quantified by qPCR using a known concentration of a serially diluted 152 bp synthetic oligonucleotide as a standard. qPCR reactions contained 1X Power Syber Green mastermix (Life Technologies, USA), 0.4 μM Ion Torrent primers A and P1, and 2 μl DNA template, and were run with the following thermal conditions: initial denaturation at 95 °C for 5 min followed by 30 cycles of denaturation at 95 °C (30 s), annealing and extension at 60 °C (45 s). Templating emulsion PCR and enrichment were performed according to the manufacturer’s recommendations on the One-Touch 2 and One-Touch ES instruments (Life Technologies, USA). Sequencing was performed on an Ion Torrent PGM (Life Technologies, USA) using 400 bp chemistry and 316-V2 semiconductor chips, following the manufacturer’s recommendations.

### Sequence processing and analysis

Sequences were first processed in Geneious 8.0.4 [74] by retaining only reads with perfect 27F-Y and 338R primers and MID sequences (no mismatches allowed). Sequences were then de-multiplexed into individual samples based on their unique combination MID sequences. Primer sequences and distal bases were trimmed from each read, and reads shorter than the minimum reported length of the amplicon (<250 bp) were discarded. Remaining reads were quality filtered using USEARCH [75], allowing only reads with a < 1 % error rate to remain and singletons were removed on a per-sample basis. In order to identify bacterial genera present in samples operational taxonomic units (OTUs) were selected by clustering sequences at 97 % similarity with the UPARSE algorithm [76]. OTUs were checked against the ChimeraSlayer Gold reference database with the UCHIME algorithm [77] to ensure OTUs were not the result of chimeric reads. Genus level taxonomy was assigned to OTUs against the GreenGenes 16S database (August 2013 release) [78] in QIIME 1.8.0 [72] using the UCLUST algorithm [75] with default parameters. Only OTUs assigned to the genus level were used for further analysis. Bacterial genera that were identified in extraction reagent blanks and no-template controls were removed from the dataset to eliminate background bacterial sequences.

16S sequences from genera that contained known tick-borne pathogens, known tick endosymbionts, or medically important bacteria that have not previously been associated with ticks, were compared against the NCBI GenBank Nucleotide database using BLAST [79] in an attempt to resolve species level taxonomy. Sequences were only assigned to a species if the query sequence matched only one species-specific reference sequence with a pairwise identity match ≥ 99 % with ≥ 99 % query coverage.

Sequences from the genera *Borrelia*, and “*Candidatus* Neoehrlichia” in this study were aligned with 16S sequences from known members retrieved from GenBank using the Geneious alignment tool [74] and refined with MUSCLE [80]. Alignments were trimmed to match the length sequences obtained in this study. *Borrelia* alignment contained 27 members with 313 bp sequences including gaps and the “*Candidatus* Neoehrlichia” alignment contained 43 members with 309 bp sequences including gaps. Neighbour-joining phylogenetic trees were constructed from these alignments in Geneious [74] using the Tamura-Nei genetic distance model and resampling 1000 bootstrap replicates. “*Borrelia*” and “*Candidatus* Neoehrlichia” sequences from “*I. holocyclus*” ticks used for phylogenetic reconstructions were deposited in GenBank (accessions KT203914-6).

## Results

### Validation of blocking primer

Comparison of unique sequences recovered from PCR amplification with or without the *Mid*Blocker primer revealed 46,698 vs. 14,154 sequences for *I. holocyclus* and 30,689 vs. 12,723 sequences for *I. ricinus*, respectively. *Ixodes holocyclus* and *I. ricinus* samples amplified without the *Mid*Blocker primer contained a total of 98.2 % and 99.6 % CMM sequences respectively, while amplification with the *Mid*Blocker primer decreased the number of CMM sequences to a total of only 2.3 % and 3.6 % of the reads respectively. Six of ten *I. holocyclus* samples and four of ten *I. ricinus* samples still contained CMM >sequences after amplification with the *Mid*Blocker primer, however, these sequences comprised < 4 % of sequences in each of these samples.

**Fig. 2:**
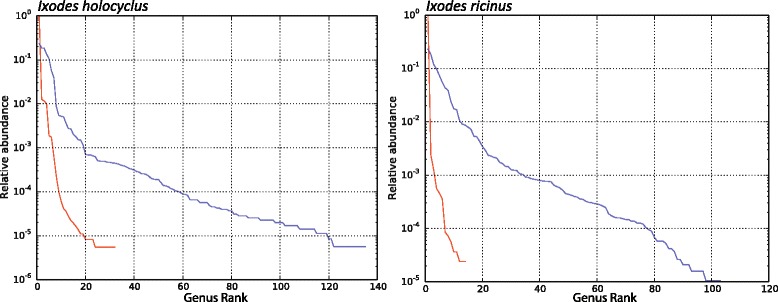
Rank abundance plots of bacterial genera identified with and without blocking. The ranked relative abundance of bacterial genera identified in 10 *I. holocyclus* and 10 *I. ricinus* ticks when amplified with (blue lines) and without (red lines) the *Mid*Blocker primer. X-axis represents the number of bacterial genera identified

Consequent to the blocking step, all samples had a significantly higher taxonomic diversity when amplified with the *Mid*Blocker primer than when amplified without the *Mid*Blocker primer (*p* < 0.05; Mann-Whitney U-Test). Amplification without the *Mid*Blocker primer resulted in the detection of 32 and 14 bacterial genera in *I. holocyclus* and *I. ricinus* samples respectively, while inhibition of CMM 16S sequences resulted in the detection of 103 and 89 additional bacterial genera in *I. holocyclus* and *I. ricinus* samples respectively (Fig. 2). Furthermore, the *Mid*Blocker primer did not appear to inhibit the amplification of other Rickettsiales closely related to CMM, as confirmed by the identification of members of the closely related *Rickettsia* and “*Candidatus* Neoehrlichia” genus in *I. holocyclus* and *I. ricinus* samples amplified with the *Mid*Blocker primer.

### Bacterial pathogens in *I. holocyclus* and *I. ricinus* ticks

After sequence processing, a total of 2,441,958 and 412,130 sequences were generated for *I. holocyclus* and *I. ricinus* ticks, respectively. Sixty-five bacterial genera were detected in extraction reagent and no-template controls, of which 28 were also present in at least one tick sample (Additional file 1). These genera were all associated with ubiquitous environmental and commensal bacteria and were subtracted from samples in order to eliminate potential environmental contaminants from the dataset. After removing background taxa a total of 199 and 95 bacterial genera were identified in *I. holocyclus* and *I. ricinus* samples, respectively (Additional files 2 and 3). Most bacteria identified were environmental and free-living bacteria often associated with soil and leaf-litter environments, characteristic of tick habitats. CMM was still the most common bacterium identified in *I. holocyclus* ticks (75.5 %) and the second most common in *I. ricinus* ticks (70 %) after *Rickettsiella* spp. However, CMM sequences comprised an average of only 6.8 % and 4.3 % of sequences per sample for *I. holocyclus* and *I. ricinus*, respectively. Six genera of medical importance were found in tick samples including tick-borne pathogens in the genera *Anaplasma*, *Bartonella, Borrelia, “Candidatus* Neoehrlichia”*,* and *Rickettsia*, and the free-living pathogens *Leptospira* and *Clostridium.*

*Bartonella henselae* was identified with 100 % sequence similarity to multiple known reference sequences [GenBank: AJ223779, HG726042, HG969191, JN646651] in one female *I. holocyclus* removed from a domestic cat. Additionally, a second *Bartonella* sp*.* was identified from a female *I. holocyclus* removed from a human. *Bartonella* sequences in this sample had multiple > 99 % matches to three *Bartonella* species *B. coopersplainsensis, B. australis,* and *B. rattaustraliani* [GenBank: EU111759, DQ538394, EU111751]; species reported to date only in native Australian wildlife. *Bartonella* species were not identified in any *I. ricinus* ticks.

**Fig. 3:**
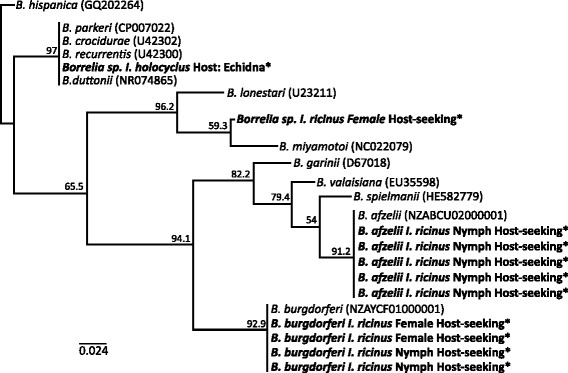
Neighbour-joining tree of 16S V1-2 *Borrelia* sequences from *I. holocyclus* and *I. ricinus* ticks. Branch labels are bootstrap values inferred from 1000 replicated. Parenthesises after node labels refers to the GenBank accession number. * Indicates sequences from this study

*Borrelia* 16S sequences were obtained from ten questing *I. ricinus* ticks and a single *I. holocyclus* tick removed from a wild Echidna (Tachyglossidae sp*.*). *Borrelia* sequences derived from the *I. holocyclus* tick had 100 % sequence similarity, and clustered with high bootstrap confidence (91.1 %) into a group of pathogenic relapsing fever *Borrelia* species including *B. duttonii, B. recurrentis, B. parkeri*, and *B. crocidurae* (Fig. 3)*. Borrelia* 16S sequences derived from one *I. ricinus* tick clustered with high bootstrap confidence (90.2 %) with the pathogenic relapsing fever *Borrelia* spp. *B. miyamotoi* and *B. lonestari*, with 99.3 % and 97.7 % sequence similarity respectively. Sequences derived from nine other *I. ricinus* ticks had 100 % sequence identity and clustered with the Lyme borreliosis-causing *B. burgdorferi* and *B. afzelii* with bootstrap values of 93.4 % and 86.8 % respectively (Fig. 3).

**Fig. 4:**
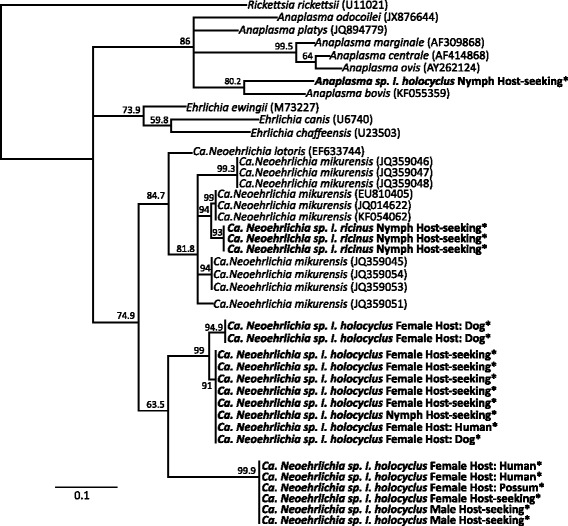
Neighbour-joining tree of 16S V1-2 “*Candidatus* Neoehrlichia” sequences from *I. holocyclus* and *I. ricinus* ticks. Branch labels are bootstrap values inferred from 1000 replicated. Parenthesises after node labels refers to the GenBank accession number. * Indicates sequences from this study

Three *I. ricinus* ticks and 15 *I. holocyclus* ticks contained sequences from the genus “*Candidatus* Neoehrlichia” and all *I. ricinus-*derived sequences had > 98 % sequence similarity, and clustered with “*Candidatus* Neoehrlichia mikurensis” reference sequences (Fig. 4). *Ixodes holocyclus-*derived “*Candidatus* Neoehrlichia” sequences formed two distinct novel clades with high bootstrap confidence (94.2 % and 97.2 %) that did not group with any “*Candidatus* Neoehrlichia” sequences in GenBank (Fig. 4). Sequences within each of these novel *“Candidatus* Neoehrlichia” clades were less than 1 % dissimilar to each other but more than 6 % dissimilar to any known “*Candidatus* Neoehrlichia mikurensis” or “*Candidatus* Neoehrlichia lotoris” 16S sequences. One *I. holocyclus* tick also contained sequences that grouped with relatively high bootstrap confidence (75.1 %) with *Anaplasma bovis* within a clade that also includes the pathogens *A. platys, A. Phagocytophilum* and *A. odocoilei* (Fig. 4).

The genus *Rickettsia* was identified in five *I. ricinus* ticks and six *I. holocyclus* ticks. In two *I. ricinus* ticks, *R. helvetica* was identified with 100 % matches to reference sequences [GenBank: L36212, KJ740388, GQ413963] and no other matches > 97 %. Four *I. ricinus* ticks were infected with *Rickettsia* spp. that could not be identified to the species level due to high sequence homology (> 99 %) between many sequences including pathogenic and benign species: one of these ticks was also co-infected with *R. helvetica. Rickettsia* sequences in six *I. holocyclus* ticks were unable to be resolved to the species level due to high sequence homology (> 99 %) at the loci sequenced between many *Rickettsia* spp., including pathogenic and benign species.

The genera *Leptospira* and *Clostridium* were identified in 18 and 30 *I. holocyclus* ticks respectively. *Leptospira* sequences derived from all ticks had 100 % sequence similarity with *Leptospira inadai* [GenBank: NR115296, AY631891, AY631887] and did not match any other species-specific sequence > 98 %. *Clostridium* sequences from 15 *I. holocyclus* ticks matched with the pathogenic *Clostridium histolyticum* [GenBank: NR113187, NR104889] with sequence similarity (99.4 %), however species designation of sequences from the 10 other ticks were unable to be resolved due to high sequence homology (> 99 %) with between many *Clostridium* spp.

### Bacterial endosymbionts in *I. holocyclus* and *I. ricinus* ticks

In addition to CMM mentioned previously, the genus *Francisella* was identified in three questing *I. holocyclus* nymphs. *Francisella* sequences from these ticks matched > 98 % with *Francisella*-like endosymbionts from *Amblyomma*, *Dermacentor*, and *Ornithodoros* ticks, and all sequences were > 6 % dissimilar from the zoonotic pathogen *Francisella tularensis.* The arthropod endosymbiotic genus *Rickettsiella* was also identified in eight *I. holocyclus* ticks and 15 *I. ricinus* ticks, however species-specific discrimination was not possible due to high sequence homology (> 99 %) between many *Rickettsiella* species at the loci sequenced. The common arthropod endosymbiont *Wolbachia* was also detected in a single *I. holocyclus* tick, which matched > 94 % to *W. pipientis* and other *Wolbachia* endosymbionts of arthropods.

## Discussion

Blocking primers are a useful tool in molecular microbiology studies, reducing amplification of overabundant sequences that would otherwise dominate sequencing results [70, 81–83]. The application here of a CMM-specific blocking primer significantly reduced the number of CMM sequences in *I. ricinus* and *I. holocyclus* samples*,* allowing identification of previously occult bacteria including other endosymbionts and potential pathogens.

Not unexpectedly, *Borrelia burgdorferi* and *B. afzelii* were detected in *I. ricinus* ticks. The prevalence of these bacteria is high in European tick populations [84] but these Lyme disease-causing agents were not detected in Australian *I. holocyclus* ticks. However, DNA of a relapsing fever *Borrelia* sp. was detected in a single *I. holocyclus* tick from a wild echidna that had 100 % identity to the known relapsing fever pathogens *B. duttonii, B. recurrentis, B. parkeri,* and *B. crocidurae.* The significance of this finding is uncertain; *Borrelia*-like organisms have been suggested in Australia previously [18, 85, 86] but this is the first report of a relapsing fever *Borrelia* species in Australia, a finding that may have public health implications. Symptoms of *Borrelia* relapsing fever can be severe, inducing fevers, myalgia, arthralgia, lethargy, petechial rash, photophobia, and facial palsy.

The organism “*Candidatus* Neoehrlichia mikurensis” is an emerging tick-borne pathogen that has been detected in rodents, humans, and ticks throughout Europe and Asia [87–90]. A second member of the genus designated “*Candidatus* Neoehrlichia lotoris” has also been described as a pathogen in the American racoon, *Procyon lotor* [91]. “*Candidatus* N. mikurensis” causes significant illness in immunocompromised humans including, but not limited to, anaemia, deep vein thrombosis, fever, diarrhoea, joint and muscle pain, pulmonary embolism, and arterial aneurysm [87–89]. Based on the partial 16S sequences reported here, the “*Candidatus* Neoehrlichia” spp. from *I. holocyclus* ticks are closely related to, but distinct from, “*Candidatus* N. mikurensis” and “*Candidatus* N. lotoris”, and may therefore be a novel species. In fact, this is the first description of the “*Candidatus* Neoehrlichia” genus in Australia; the medical significance of this finding warrants further research to refine its phylogenetic position and investigate its pathogenicity, if any, in humans. Furthermore, the detection of an *Anaplasma* sp. in one *I. holocyclus* tick is also of significance, as only two species of *Anaplasma* have previously been detected in Australia; *Anaplasma marginale* in *Rhipicephalus microplus* ticks [92], and *Anaplasma platys* in *R. sanguineus* ticks in central and northern Australia [93].

Detection of *Leptospira inadai* during this study may explain the observation over twenty years ago of spirochaete-like objects (SLOs) identified by dark field microscopy of various tick species including *I. holocyclus* [17]*.* Although these SLOs were dismissed as aberrant artifacts by the authors, it is noteworthy that the SLOs shown in Figs. 1 and 2 from Russell et al. [17] bear a strong resemblance to various *Leptospira* spp., including *L. inadai*. Further work isolating and imaging *L. inadai* from *I. holocyclus* is required to confirm this possibility. Recently it was proposed that *Leptospira* spp. may also be tick-transmitted due to their high prevalence in *I. ricinus* ticks [94]. *Leptospira inadai* is pathogenic in laboratory rodents and *L. inadai* serovar Lyme was isolated from a skin biopsy of a human Lyme disease patient in North America [95]. Although in that case *L. inadai* was not thought to be associated with the patient’s symptoms, its high prevalence in *I. holocyclus*, and the high prevalence of *Leptospira* spp. in *I. ricinus* warrants further investigation.

*Francisella*-like endosymbionts are well described in *Amblyomma* and *Dermacentor* ticks, and have recently been detected in *I. ricinus* ticks [28, 29, 96]. In this study we report the first instance of a *Francisella* sp. in the Australian paralysis tick *I. holocyclus.* Many *Francisella*-like endosymbionts infect tick species that are also capable of transmitting the zoonotic pathogen *Francisella tularensis,* making accurate identification by conventional PCR methodologies challenging due to false positive results [97]. The methodology presented here accurately identified non-tularaemia-causing *Francisella* spp. endosymbiont 16S sequences that were 6 % dissimilar from *F. tularensis* reference sequences, indicating that NGS and bioinformatics methodologies may prove useful in clinical diagnostic settings.

## Conclusions

Next-generation 16S bacterial profiling is an excellent tool for the simultaneous identification of many bacterial species in arthropods. However, bacterial endosymbionts such as CMM, which are common and abundant in many arthropod vectors such as ticks and mosquitoes, can limit the effectiveness of this method by biasing PCR amplification of less abundant sequences. Here we have shown that a CMM-specific blocking primer significantly increases the amplification and detection of less abundant bacteria including pathogens. Furthermore our CMM-blocking primer is applicable to a range of arthropods that harbour CMM, and can be applied to a wide variety of disease vectors.

In this study we identified novel candidate pathogens that warrant further scrutiny in the context of investigating so-called “Lyme-like disease” in Australia. *Borrelia* relapsing fever and “*Candidatus* Neoehrlichia” pathogens are being identified in new geographic regions throughout the world and their medical importance is well recognised. The aetiological agent of Australian “Lyme-like” illness has been a source of unresolved debate for many years and the discovery of these organisms in Australian *I. holocyclus* ticks may provide insights into this medical conundrum. Given the widespread presence of endosymbionts in arthropod vectors of disease, together with the fact that such symbionts may be resident in high numbers, our findings also highlight the potential for discovering important novel arthropod-associated bacteria that are in relatively low abundance.

## Additional files

Additional file 1:
**Cladogram of bacterial genera identified in extraction reagent blank and no-template controls.**
Additional file 2:
**Cladogram of bacterial genera identified in** ***I. holocyclus*** **tick samples after the removal of genera found in control samples.** Size of node circle represents the relative abundance of that genus between tick samples on a square-root scale. Additional file 3:
**Cladogram of bacterial genera identified in** ***I. ricinus*** **tick samples after the removal of genera found in control samples.** Size of node circle represents the relative abundance of that genus between tick samples on a square-root scale.
