# Supplementary figures and images for: Inhibition of the endosymbiont “Candidatus Midichloria mitochondrii” during 16S rRNA gene profiling reveals potential pathogens in Ixodes ticks from Australia
Source: Parasit Vectors. 2015 Jun 25;8:345. doi: 10.1186/s13071-015-0958-3 (PMC4493822; doi:10.1186/s13071-015-0958-3)

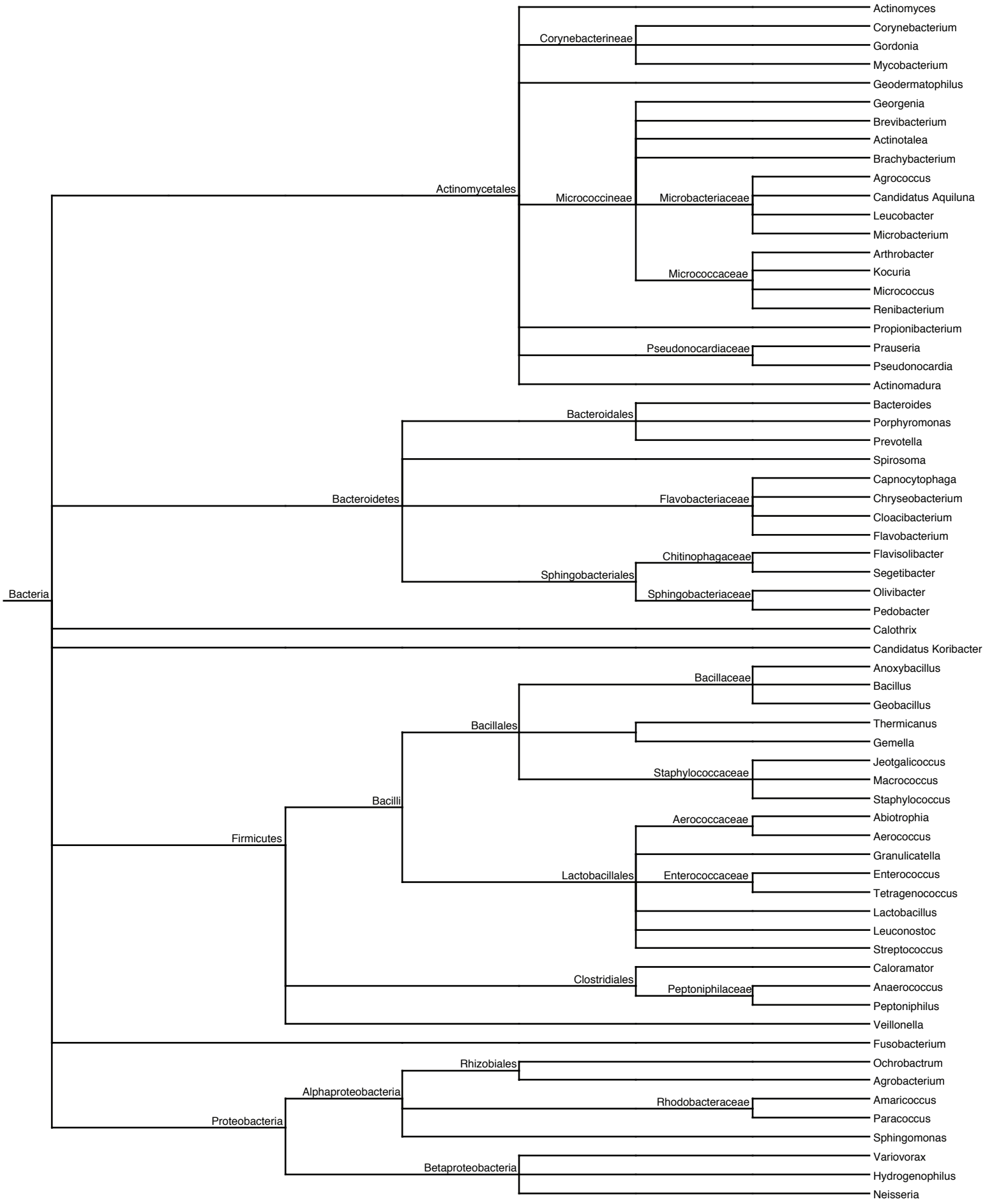

Supplement: Additional file 1: — Cladogram of bacterial genera identified in extraction reagent blank and no-template controls. [file 13071_2015_958_MOESM1_ESM.pdf]

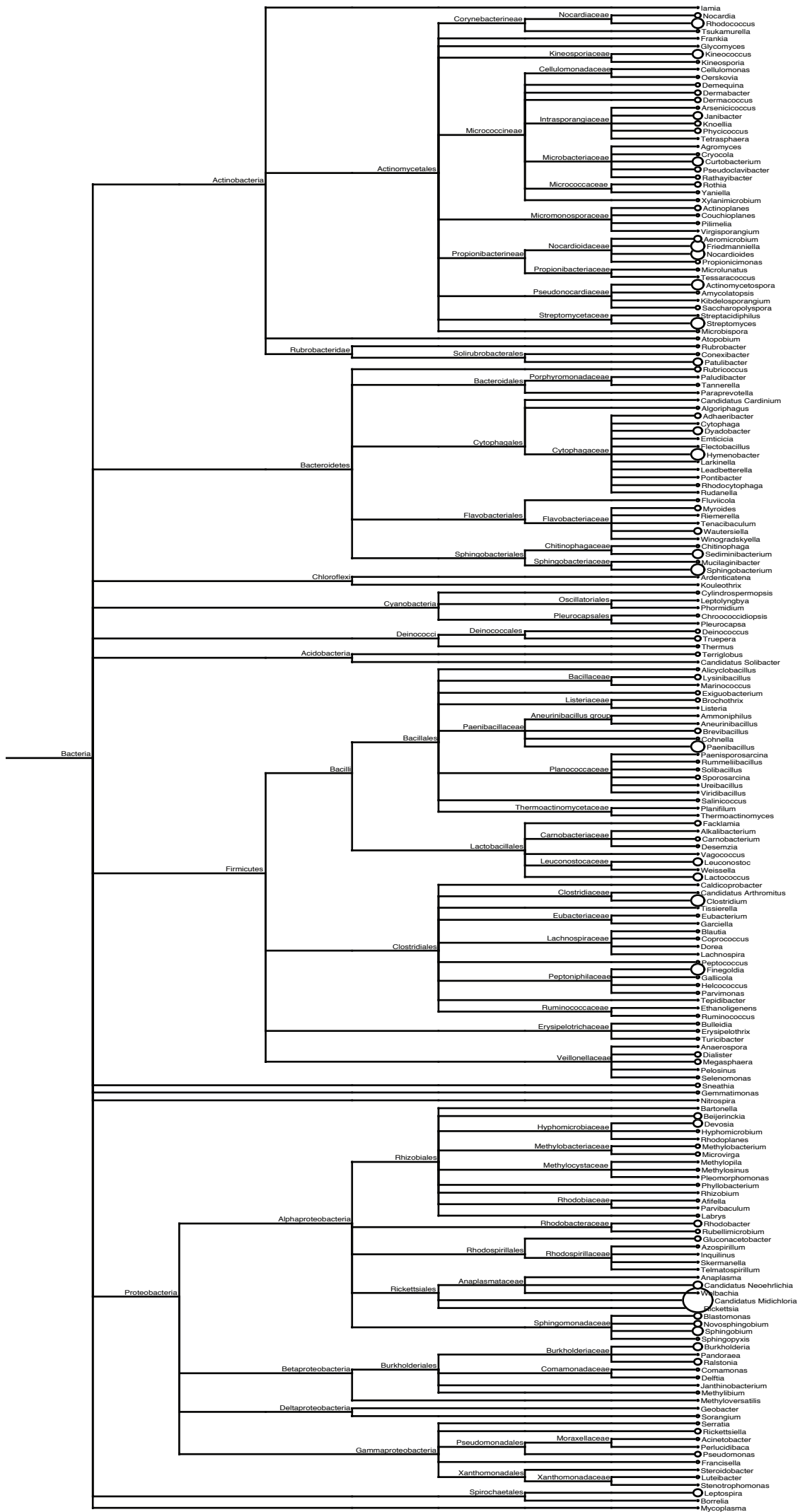

Supplement: Additional file 2: — Cladogram of bacterial genera identified in I. holocyclus tick samples after the removal of genera found in control samples. Size of node circle represents the relative abundance of that genus between tick samples on a square-root scale. [file 13071_2015_958_MOESM2_ESM.pdf]

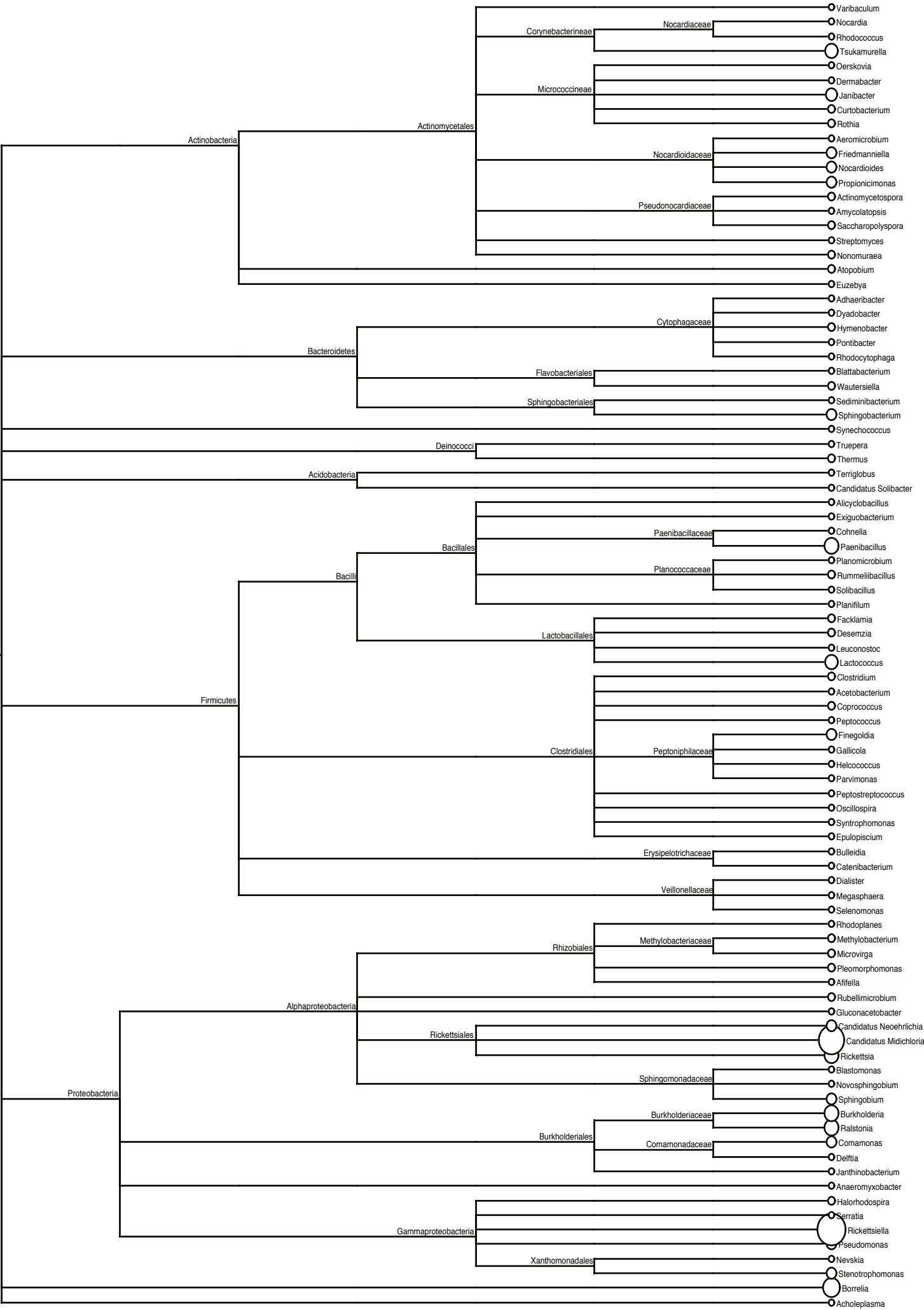

Supplement: Additional file 3: — Cladogram of bacterial genera identified in I. ricinus tick samples after the removal of genera found in control samples. Size of node circle represents the relative abundance of that genus between tick samples on a square-root scale. [file 13071_2015_958_MOESM3_ESM.pdf]
